# Fluoridation of a lizard bone embedded in Dominican amber suggests open-system behavior

**DOI:** 10.1371/journal.pone.0228843

**Published:** 2020-02-26

**Authors:** H. Jonas Barthel, Denis Fougerouse, Thorsten Geisler, Jes Rust

**Affiliations:** 1 Paleontology Section, Institute of Geosciences, RheinischeFriedrich-Wilhelms-Universität Bonn, Bonn, Germany; 2 School of Earth and Planetary Sciences, Curtin University, Perth, Australia; 3 Geochemistry/Petrology Section, Institute of Geosciences, RheinischeFriedrich-Wilhelms-Universität Bonn, Bonn, Germany; Monash University, AUSTRALIA

## Abstract

Vertebrate fossils embedded in amber represent a particularly valuable paleobiological record as amber is supposed to be a barrier to the environment, precluding significant alteration of the animals’ body over geological time. The mode and processes of amber preservation are still under debate, and it is questionable to what extent original material may be preserved. Due to their high value, vertebrates in amber have never been examined with analytical methods, which means that the composition of bone tissue in amber is unknown. Here, we report our results of a study on a left forelimb from a fossil *Anolis* sp. indet. (Squamata) that was fully embedded in Miocene Dominican amber. Our results show a transformation of the bioapatite to fluorapatite associated with a severe alteration of the collagen phase and the formation of an unidentified carbonate. These findings argue for a poor survival potential of macromolecules in Dominican amber fossils.

## Introduction

Fossil bones represent valuable paleontological archives for reconstructing the paleobiology and -environments of vertebrates throughout geological time and thus also represent an important window into the evolution of life on Earth. The preservation of organisms or parts of them over long geological timescales requires exceptional conditions before and after death of the organism. During diagenesis, the remains are affected by various chemical processes like dissolution or pseudomorphosis, so the original material, especially the organic soft tissue, is often lost or severely modified. However, a detailed understanding of the preservation and fossilization of bone at the microscopic scale is still lacking. This is partly because bone is a complex hierarchical composite material. It consists of a nano-crystalline, hydrated, hydroxylated, and carbonated calcium phosphate phase (hydroxylapatite (HAp)-like) that is embedded in a fibrous organic matrix of predominately collagen and subordinately lipids. A recent vibrational spectroscopic study [1] suggested that molecular water is a stabilising component of biogenic apatite (bioapatite), which has also been postulated in previous studies based on nuclear magnetic resonance spectroscopy [2]. Pasteris et al. [1] proposed that the chemical formula of bioapatite should be Ca_10–x_[(PO_4_)_6–x_(CO_3_)_x_] (OH)_2–x_ · nH_2_O, where n ≈ 1.5 and x ranges between 0.1 and 0.3. The OH group in bioapatite can be replaced by F, whereas Ca may partly be substituted by, e.g., Mg, Zn, Sr, Na, and K [3]. Fluorine was found of particular importance for the preservation of bone and teeth during diagenesis [4, 5] as well as for caries prevention by transforming HAp to more stable, i.e., less soluble fluorapatite (FAp) [6]. Bone apatite is thus a complex solid solution that occurs as nano-crystals with sizes in the order of 20 to 150 nm. Due to its crystal-chemical properties, bioapatite is a highly reactive phase that, if its physicochemical environment is changing (for instance, after death of the organism), has a high thermodynamic driving force to dissolve [4] or to react in aqueous solutions that are (super)saturated with respect to apatite. Under certain conditions original bone tissues survive over geological time scales, includingorganic components (e.g., collagen) that could still be detected in dinosaur bone [7, 8]. Such bone specimens are often characterised by a larger average crystallite size, a higher crystallinity, and a different apatite chemistry with respect to the original bone [9–12].

The entrapment of an organism in viscous tree resin is a unique prerequisite in terms of fossilization, with the chance to preserve embedded organisms in a three-dimensional, life-like posture. In public perception, amber is therefore often referred to as a “time capsule,”prohibiting the majority of decay processes. Amber represents a strong taphonomic filter and favors the conservation of small organisms such as insects and spiders, which are often extremely well preserved with ultrastructural detail [13, 14, 15]. The liquid resin initially protects entrapped organisms from microbial attack and predators, which represents an important basis for preservation. However, evidence has been reported showing that even air can pass through amber, which may cause oxidation reactions [16]. In general, it is well known that the preservation of fossils in amber differs largely throughout specimens and amber deposits. The degree of preservation ranges from the relict occurrence of straight chain hydrocarbons and altered macromolecules of beetles in Dominican amber to still reacting cellular components of cypress twigs in Baltic amber [17, 18].

Compared to the large amounts of arthropods reported as inclusions in amber, only a small number of vertebrate remains of frogs, lizards, birds, mammals, and dinosaurs has been reported from different amber deposits from the Cretaceous to the Neogene around up to 120 million years old [19–27]. Most of these findings refer to small arboreal lizards of the family Gekkonidae and the genus *Anolis*, comprising partial remains to complete specimens [28–38]. X-ray scans revealed that parts of the skeleton are preserved in most of these specimens [28, 39, 40], but despite this observation, nothing is known about the degree of preservation of bone material in amber. One obvious hypothesis is that bones that were embedded in amber and thus shielded against aqueous solutions, may show a high degree of preservation, including their collagen matrix.

**Fig 1:**
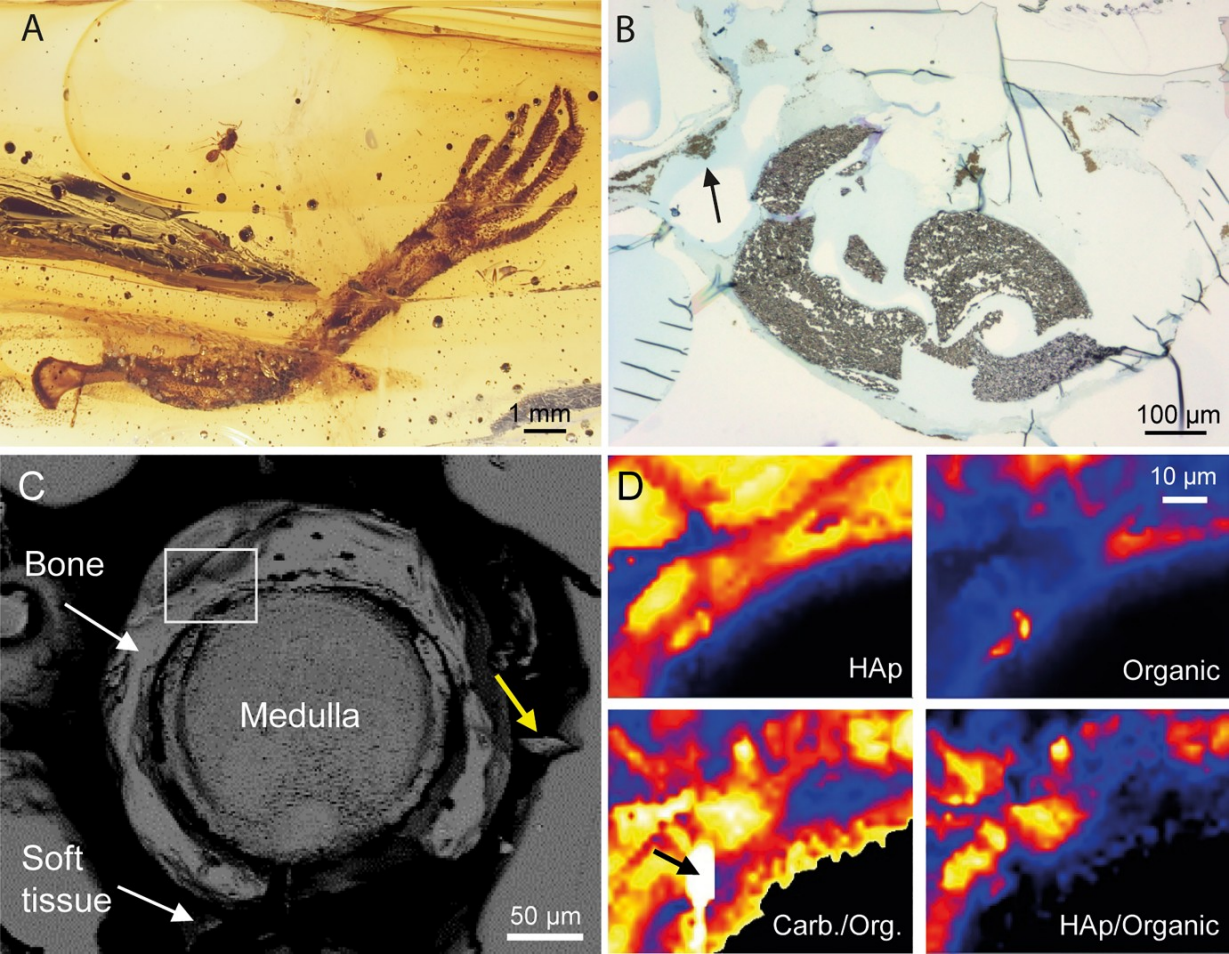
**(A)** Light microscopic image of the investigated sample DHQ-4924-H. The piece contains a fairy wasp and the left forelimb of an anole lizard. Several flow structures can be recognized in the resin. **(B)** Optical image of a microtome slice of the fossil humerus, showing fragmented bone tissue (dark in the image). During the cutting of the sample, the fragile bone broke because of the increasing tension in the material. The black arrow points to epidermal remains in the resinous matrix. **(C)** Backscattered electron image of the fossil bone surface. The yellow arrow shows a bone fragment that was pressed into the material during preparation of the sample. The white rectangle marks the area that was imaged by Raman spectroscopy: **(D)** False-coloured hyperspectral Raman images of a bone area, showing (**upper left**) the integrated intensity of the ν_1_(PO_4_) apatite band (A_ν1(PO4)_), (**lower left**) the intensity ratio of the ν_1_(CO_3_) band of carbonate near 1070 cm^-1^ (A_ν1(CO3)_) and the total intensity between 1150 and 1700 cm^-1^, reflecting the organic content (A_1150-1700_), (**upper right**) A_1150-1700_, and (**lower right**) A_ν1(PO4)_ / A_1150-1700._ An unknown carbonate phase is marked by a black arrow.

To address this question, we examine the left forelimb of an *Anolis* sp. indet. in a piece of 15 to 20 Million years old Dominican amber, also including a fairy wasp (Mymaridae, Fig 1A and S1 Fig), by micro-Raman spectroscopy, electron microprobe, and time-of-flight secondary ion mass spectroscopy (ToF-SIMS). A detailed description of the fossil specimen is provided in the Supporting Information (S1 Appendix, S1–S3 Figs).

Microtome cuttings of the fossil bone reveal a brittle tissue that might be attributed to contact with acidic resin compounds shortly after entombment. The alteration of the bone is confirmed by our spectral Raman data, and electron microprobe measurements, which indicate a transformation of the original bioapatite into fluorapatite. Furthermore, the collagen matrix is largely degraded to possibly C-H-C aliphatic compounds. Measurements in the bone cavity revealed complex spectra that have to be interpreted in the future and are likely the product of epoxy resin compounds and original material. We discuss several possible sources of the fluorine but expect an allochthon origin to be most likely, based on our ToF-SIMS analysis. Although we cannot explain the bone fluoridation and alteration in detail, it is important to note that evidently the presence of a resinous matrix does not necessarily inhibit chemical exchange between fossil and the environment of the amber. Therefore, vertebrate fossils in amber must be considered as the result of open-system processes which severely limits the potential of original macromolecular compounds.

## Results

**Fig 2:**
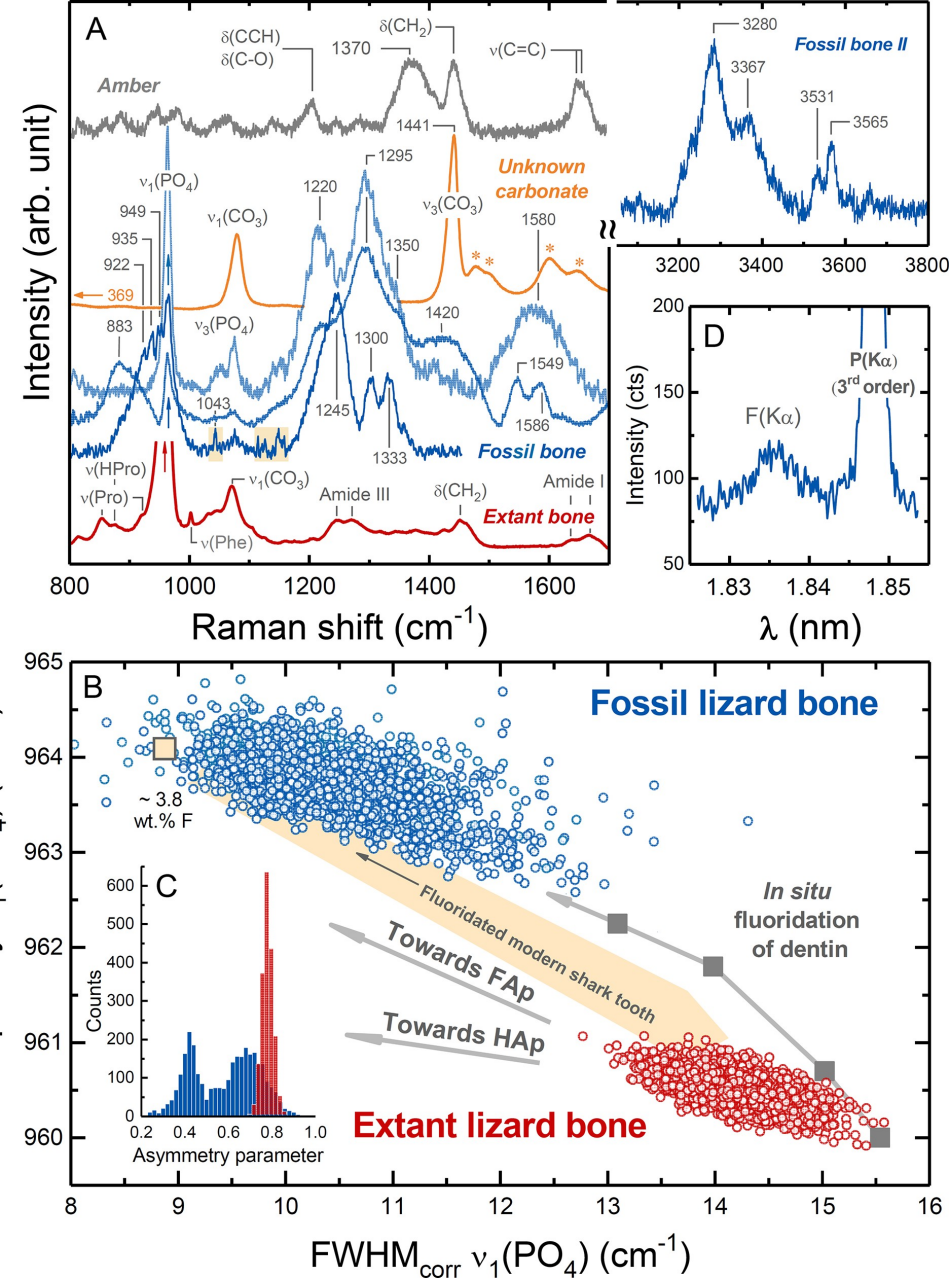
**(A)** Raman spectra of the fossil bone (blue), extant bone (red), the unknown carbonate (yellow) and the amber matrix (grey). **(B)** The relationship between the width, given as full width at half (FWHM), and frequency of the ν_1_(PO_4_) mode for bone apatite shows that the fossil bone (4,323 analyses) is associated with higher frequencies and overall smaller FWHM values compared to the extant sample (2,527 analyses). In comparison with data from an extant shark tooth and experimental bone fluoridation data [49], this observation suggests the formation of fluorapatite. **(C)** The distribution of the asymmetry parameter of the ν_1_(PO_4_) band of fossil and extant bone spectra. The asymmetry parameter of the fitted asymmetric Gauss-Lorentz function varies between -1 and +1 with 0 referring to a symmetric profile. Negative values skew the spectrum toward higher while positive values, as observed here, skew it toward lower wavenumbers. The multimodal distribution observed for the fossil bone indicates variable degrees of crystallinity and/or crystallite size. **(D)** An electron microprobe wavelength-dispersive X-ray scan from the fossil bone, which further verifies the occurrence of F in the fossil bone. See text for further discussions.

After light microscopic and μ-CT examinations, the sample was cut and polished so that a portion of the humerus was exposed and polished perpendicular to its longitudinal axis (Fig 1C). Raman spectroscopy was then used to first characterize the embedding amber close to the fossil. The most obvious features in the amber spectrum (Fig 2A) are the (fingerprint) bands at 1644, 1660, and 1450 cm^−1^, resulting from the exomethylene stretching ν(C = C) and the scissoring type deformation δ(CH_2_) vibration in the resin structure, respectively [40, 41, 42]. It has been demonstrated that the integrated intensity ratio of the doublet near 1650 cm^-1^ and the band at 1450 cm^−1^ is a good indicator of the maturity of fossilized resins. In general, the ratio is >1 for immature resins such as copal and < 1 for mature (fossil) resins such as amber [41, 43]. From a least-squares fit of Gauss-Lorentz functions to the three bands along with a polynomial background, we obtained a ratio of 0.92 ± 0.09 for our sample that agrees very well with published values from Dominican amber which range between 0.91 and 1.00 [41]. A noticeable difference with published amber spectra, however, is the occurrence of a broad band near 1370 cm^−1^. Moreover, the bands at 1355, 1384, and 1408 cm^−1^, assigned to δ(CH_2_) and δ(CH_3_) deformation modes, are significantly more intense, even reaching the same height as the neighboring ν(C = C) band at 1450 cm^−1^. These spectral differences indicate that the amber has chemically been modified.

The generally good preservation property of the amber is noticeable by the conservation of skin soft tissue that caused problems with the polishing of the bone due to the difference in the hardness between amber, tissue, and bone, so that the sample surface is not perfectly flat (Fig 1C). However, the bone itself also exhibited brittle behaviour during polishing as reflected by cracks and broken bone pieces that are partly pressed into the surrounding amber (yellow arrow in Fig 1C), indicating that some alteration occurred that affected the mechanical properties of the bone. This observation is further confirmed by the histological slices of the fossil. Except for one slice (Fig 1B), the fossil bone tissue was highly fragile and was lost from the microscope slices during preparation. This can be explained by an extensive fragmentation of the bone tissue (Fig 1B) that we expect to stem from the contact with acidic compounds in the resin. Unexpectedly, hyperspectral Raman images from a 50 x 50 μm^2^ area of the bone further revealed an unusually heterogeneous distribution of organic and inorganic phases (Fig 1D), including the occurrence of a new, yet unidentified carbonate mineral (black arrows in Fig 1D). This phase is unambiguously identified as a carbonate by the ν_1_(CO_3_) band near 1079 cm^‒1^ (Fig 2A) and shows only two further Raman bands near 369 and 1441 cm^‒1^, the latter of which can be assigned to the antisymmetric ν_4_(CO_3_) stretching motion of the carbonate group. All three bands are relative broad with linewidths between 20 and 30 cm^−1^ (Fig 2A), suggesting a rather disordered carbonate structure. Unfortunately, its Raman spectrum is not comparable to any of the known (carbonate) minerals that are included in the RRuff Raman spectral data base containing more than 8000 Raman spectra of minerals [44].

From a comparison of representative Raman spectra of the fossil bone with a typical Raman spectrum of an extant lizard bone it is evident that both the inorganic and organic phases in the fossil bone are strongly modified (Fig 2A). In the Raman spectra of the extant bone, bands related to collagen vibrations, such as methylene side chains (CH_2_ at 1450 cm^−1^), amide III (1243–1320 cm^−1^), phenylalanine (~ 1002 cm^−1^), CH stretch (~ 2940 cm^−1^), or the combined proline (921 and 855 cm^−1^) and hydroxyproline (876 cm^−1^) vibrations, can clearly be identified (Fig 2A). In contrast, Raman spectra from the fossil do not show any of the collagen bands anymore, but are characterized by a number of new broad bands between 1200 and 1500 cm^−1^ which can be assigned to ν_s_(C−O) and/or ν_s_(C−N) vibrations. In some spectra, sharper but weak bands can be identified at 1043, 1115, 1125, and 1148 cm^−1^ (marked yellow in Fig 2A). These spectra also show overlapping bands near 922, 935, and 949 cm^−1^, whereas in other spectra a broad band near 883 with a shoulder near 920 cm^−1^ is visible in the wavenumber range between 800 and 1000 cm^−1^, i.e., at typical frequencies of aliphatic C−C chain vibrations. Some spectra show a broad band near 1580 cm^−1^ and a shoulder near 1350 cm^−1^ (Fig 2A) that may tentatively be assigned to the D_1_ and G band of amorphous sp^2^ carbon, respectively [45]. However, the spectra vary in different locations with some spectra showing two distinguishable bands near 1549 and 1586 cm^−1^ rather than a broad band centred near 1580 cm^−1^, which possibly reflect aliphatic ν(N = N) modes. Noticeably, no Raman signals were detected in the frequency region between 1650 and 3100 cm^−1^, suggesting the absence of, e.g., aliphatic C‒H bonds and carboxyl functional groups. Relative weak bands could be detected in the fossil bone between 3200 and 3600 cm^−1^, indicating the occurrence of O−H and/or N−H bonds. It follows from these observations that regardless of the exact composition and structure of these organic compounds, they obviously do not represent typical collagen degradation products such as polypeptides, smaller peptides, or amino acids.

The most intense and sharpest band in the spectra from the extant lizard bone is located near 960 cm^-1^ and reflects the fully symmetric ν_1_(PO_4_) stretching motions of the phosphate tetrahedra in the bone apatite structure (Fig 2A). The carbonate group in the apatite structure generates a ν_1_(CO_3_) band near 1070 cm^−1^ [46] that is, however, partially overlapped by ν_3_(PO_4_) stretching bands, making a quantitative assessment of the carbonate content challenging [47, 48]. All these apatite-related bands also occur in the Raman spectra of the fossil bone. However, here the ν_1_(PO_4_) band is (i) significantly shifted to higher frequencies, (ii) narrower, (iii) significantly less intense when compared to the Raman intensities from the organic material, and (iv) on average less asymmetric (Fig 2C). To quantify these visible differences, we fitted an asymmetric Gauss-Lorentz function to the ν_1_(PO_4_) band along with a 3^rd^ order polynomial background between 930 and 990 cm^-1^ to account for the asymmetry of this band, basically caused by a contribution from a band near 950 cm^−1^ of still unknown origin [49], and any non-linearity in the background of some spectra, respectively. The data from both specimens define non-overlapping, linear arrays in the plot of the frequency of the ν_1_(PO_4_) band versus its full width at half maximum (FWHM), with fossil bone data covering a larger range of frequency and FWHM values (Fig 2B). This and the broad multimodal distribution of the asymmetry parameter (Fig 2C) suggest a heterogeneous bone microstructure that is composed of domains showing variable structural disorder and/or crystallite size. Thereby, the frequency and FWHM values from the fossil bone are significantly higher respectively lower than those from the extant bone and cover values that are typical for highly fluoridated bones and teeth in which the bone apatite is transformed to FAp (Fig 2B) [49]. Due to the significance of such an observation, the occurrence of F was further verified by wavelength-dispersive X-ray scans that unambiguously verify the occurrence of F in the bone (Fig 2D).

Now the question arises about the source of the fluorine. In this respect, the occurrence of a crack in the amber piece is an important observation (S2 Fig), as it potentially represents a favored pathway for chemical transport. As determined by μ-CT scans, radius and ulna of the lizard are broken in the same plane of the crack (S1 and S2 Figs), which suggests cracking after the resin has cured and thus completely polymerized. To find evidence of chemical exchange along the crack, we analyzed this structure with focused ion beam based time–of-flight secondary ion mass spectrometry (FIB ToF-SIMS). The crack is filled with residues of epoxy resin and an aluminosilicate mineral phase (S4 Fig). ToF-SIMS analyses confirmed the presence of O, Mg, Al, Si, K and Na as major elements in the mineral. Traces of F can be observed as elongated features, likely in the cleavage planes of the mineral phase (S4 Fig). The epoxy resin is clearly a preparation artefact, and was used to stabilize the sample prior to the cutting. The epoxy is enriched in O and Cl compared to the host amber and although enrichment of Cl can be observed between the mineral phase and the amber, it remains unclear if this is the result of the epoxy contamination or is indicative of fluid infiltration (S4 Fig).

## Discussion

Although optical microscopy and μ-CT imaging revealed a high degree of morphological preservation of the fossil fore limb (S2 and S3 Figs), our Raman and X-ray data (Fig 1) from the bone surprisingly demonstrate the unambiguous formation of FAp in the fossil lizard bone. Fluoridation of bone is a process that involves the transformation or replacement of the carbonated bone HAp to a FAp structure. Fluoridation is commonly observed in fossils from clastic sedimentary environments, where it takes place during an early diagenetic stage by the infiltration of F-rich ground or soil water, and is accompanied by a simultaneous loss of the structurally incorporated carbonate groups and collagen matrix [50, 51], i.e., fluoridation is usually a *post mortem* process. However, teeth and bones may also fluoridate during the lifetime of the individual [51, 52]. Replacement of OH^‒^ by F^‒^ in teeth enamel can have the advantage of producing a harder more resistant enamel. For this reason, F has been added to municipal water supplies in some countries (USA, UK, Australia) as well as to toothpaste [53]. Experiments have confirmed that nanocrystalline hydroxylapatite in dental enamel can be replaced by fluorapatite even at room temperature within laboratory time scales [49]. It has been proposed that the replacement is pseudomorphic with the original morphology of the hydroxylapatite crystallites preserved [49].

The detection of F along the crack in the amber piece (S2 Fig) suggests that F could have been transported as F^‒^ species dissolved in water along the crack that extends to the lizard bone, but it remains unclear in which stage of diagenesis this could have happened. The polymeric and unipolar nature of the fresh and matured resin structure must have generally shielded the lizard bone against the infiltration of free water in an early diagenetic phase. The presence of flow patterns in the amber piece (S1 Appendix and S1 Fig) points to an accumulation of resin on the outer surface of the bark of the tree, and not within the bark, wood, or root area. Consequently, the piece was exposed to air, rain water, and UV-radiation before it was buried in the soil where it may have been in contact to soil or ground water. The fossil humerus is broken in its middle part (S2 Fig) and the formation of an edema (S2B Fig) in this region implies that the resin was still deformable at this time. This interpretation is supported by peeled off parts of the skin “floating” in the matrix around the bone (S1 and S2 Figs). Furthermore, an edema indicates that the lizard was not dehydrated or dead when it entered the fresh tree resin (S1 Appendix), which means that early decay processes of the fossil took place while it was fully embedded within a viscous, non-polymerized resin that included gas bubbles (S1 and S3A Figs), which prevented the escape of the body fluid. It is thus possible that internal body fluids may have played an important role by altering the mineral and organic phases of the mineralized and non-mineralized tissue.

However, it seems unlikely that the fluoridation is solely caused by body fluids, because in this case fluorine must have been locally concentrated in the liquid phase. An autochthonous origin of F may derive from an uptake of F-rich drinking water by the living lizard. In this case, the animal would have suffered from severe fluorosis before it died. Fluoride can be found in the lithosphere and natural waters in varying concentrations, with high amounts being reported from sediments of marine origin in mountainous areas, volcanic rocks, and granitic and gneissic rocks [54, 55]. With the exception of the large lakes in the East African Rift Valley system, high concentrations of F are usually associated with ground waters and not surface waters [54]. Generally, rivers and lakes have a fluoride concentration of less than 0.5 mg l^-1^. Seawater contains 1.0 mg l^-1^ of fluoride, whereas the concentration in drinking water (ground water) can reach up to 50 mg l^-1^ [54]. If no specialized structures for water uptake are present, most lizards lick water from wet leaves or drink from small ponds or rainwater. These surface waters have a low (if any) concentration of fluorine which means that their effect on the bone tissue should be negligible. In theory, another source of autochthonous fluorine can be found in the dentition of the animal, but the forelimb was separated from the body, which excludes this possibility in our case.

## Conclusions

Although at this stage of investigation the source of F remains somewhat speculative, we expect an allochthon origin to be most likely based on our ToF-SIMS analysis. However, regardless of the exact mode of fluoridation, the alteration process did not alter the bone on a large scale because lacunae of osteocytes are still visible (S3D Fig), which excludes an extensive remodeling of the structure. Furthermore, Raman spectroscopy revealed that the transformation of the nanocrystalline, carbonated bone HAp to FAp was accompanied by severe alteration of the collagen matrix, possibly to aliphatic C-N-H compounds. The intergrowth of the mineral and organic phase in bone suggests that the degradation of collagen must have taken place at the same time as the recrystallization of the carbonated bone HAp to FAp, which apparently releases carbonate groups into the surrounding solution which locally precipitated as a yet unidentified carbonate phase. In nature the chemical breakdown and loss of collagen may occur slowly in soil or groundwater via pH-dependent hydrolysis reactions (proteolysis), producing smaller peptides and amino acids, or later through conditions in a burial environment, where the degradation of collagen may increasingly be controlled by temperature [56]. In archaeological samples of Holocene bones, microbial proteolysis has been identified as the main process of collagen degradation and loss [57]. This pathway is unlikely for our sample due to the antiseptic properties of organic compounds in tree resins such as non-polymerized diterpenoids. However, a previous study focusing on ancient amino acid preservation of insects in amber [58] failed to detect any remains in the samples from Dominican amber, which is in contrast to other deposits and indicates a protein-degrading environment. On the other hand, volatile resin compounds such as mono-, and sesquiterpenoids may have already reacted with the collagen matrix during entrapment of the lizard, i.e., during the early polymerization and solidification process, but without any significant mass loss as indicated by the histological slice (Fig 1B). Some monoterpenoids and sesquiterpenoids possess enzyme inhibiting properties, which might contribute to the generally observed exceptional preservation of soft tissue in amber.

Although the exact mechanism of bone fluoridation and alteration remains unresolved at this stage, it is important to note that evidently the presence of a resinous matrix (later forming copal and amber) does not necessarily inhibit chemical exchange between fossil and the environment of the amber. As a result, fossil inclusions of vertebrates in amber must be considered as the result of complex (open-system) transport and reaction processes involving the interaction of bone tissue with (i) external chemical elements and substances, including aqueous solutions, (ii) the aqueous body fluid of the fossil itself, and (iii) highly reactive, organic resin compounds. As this is the first detailed study on fossil bone material embedded in amber, future studies on different vertebrate inclusions in amber from different deposits will show which of the above processes dominate and whether there is still a chance to find intact macromolecules in amber fossils.

## Material & methods

### Sample preparation

The investigated piece of Dominican amber is part of the paleontological collection of the State Museum of Natural History Stuttgart (SMNS, collection number DHQ-4924-H). All necessary permits were obtained for the described study, which complied with all relevant regulations. The piece contains a Mymaridae (fairy wasps, Hymenoptera) and the left forelimb of an *Anolis* sp. indet. The sample has been covered with two glass sheets on either side which have been fixed with some glutinous material (maybe Canada balsam) by its former owner. We removed the glass with a grinding machine and embedded the whole sample again in epoxy resin (Araldite2020^©^). Documentation took place between consumptive preparation steps and comprises μ-CT scans and optical images taken at different magnifications. We then cut the specimen in half, resulting in a piece containing the head of the humerus and a piece with the distal part of the specimen, including humerus, radius, ulna, and manus from which a polished thin section was prepared. In addition to the fossil specimen, we obtained a dead *Anolis carolinensis* (56 mm snout-vent length) from a private breeder. For comparison, the left forelimb (length: 15 mm) was separated from the trunk with a scalpel and then processed in the same way as the fossil specimen to produce a polished thin section. Raman spectroscopy was then used to first characterize the polished thin sections of the fossil and the recent lizard (Fig 1C).

Furthermore, we used the other half of the sample (including only the head of the humerus) to produce histological thin sections with a microtome, following roughly the same protocol as for extant specimens. An exception was the infiltration of the sample with artificial resin with the help of a desiccator. This was done to seal void spaces in the matrix that would have caused problems during the cutting.

### Optical imaging

The images of the osteocyte lacunae and thin sections were taken with a Leica DMLP polarizing microscope and the software Image Access. The lacunae are recognizable in light microscopic images, but they are better visible in polarized images.

### Micro-CT (computed X-ray tomography)

The sample was scanned with a General Electronis v‒tome‒xs micro-CT at the Institute of Geosciences, University of Bonn, Germany. The amber piece was irradiated at a voltage of 105 kV and a current of 105 mA with an exposure time of 1000 ms with a voxel size down to 11.15 μm^3^.

### Raman spectroscopy

Raman measurements were performed with a Horiba Scientific LabRam HR800 confocal Raman spectrometer at the Institute of Geosciences, University of Bonn, Germany. Raman scattering was excited with a diode-pumped solid-state laser (783.976 nm) with less than 20 mW at the sample surface. A 100 times objective with a numerical aperture of 0.9 and a confocal hole of 1000 μm was used for all measurements, resulting in a theoretical, diffraction-limited lateral and axial resolution of ~1 μm and less 1 μm, respectively. The scattered Raman light was collected in a 180° backscattering geometry by an electron-multiplier charge-coupled device detector after having passed through a 200 μm spectrometer entrance slit and being dispersed by a grating of 600 grooves/mm. This yielded a spectral resolution of 2.07 cm^‒1^, as determined by the full width at half maximum of Ne lines that were continuously monitored by placing a Ne lamp inside the beam path of the scattered light. The Ne lines were used to correct for any non-linear spectrometer drift during long term mapping. To correct for the effect of the finite slit width on measured linewidth (given as full width at half maximum, FWHM) following equation was used^(Tanabe & Hiraishi 1980)^: FWHM_c_ = FWHM_m_[1 ‒ (*S* / FWHM_m_)^2^], where FWHM_c_, FWHM_m_, and *S* are the corrected linewidth of the Raman band, the measured linewidth, and the spectral slit width (= 2.07 cm^‒1^), respectively. All spectra were further corrected by a wavelength-dependent instrumental response function (white light correction). The measurements were performed in the point-by-point mapping mode at different locations of the bone. The total acquisition time of a single spot analysis was between 60 and 300 s.

### Electron microprobe (EMP)

To further check the occurrence of F in the bone apatite, suggested by the Raman data, the sample was coated with a thin layer of gold and wavelength-dispersive X-ray spectra were recorded with a JEOL 8200 Superprobe and a TAP crystal in the wavelength range between 1.82 and 1.86 nm, in which the F-Kα line (1.832 nm) is located. The measurements were performed with a step size of 9.2 x 10^−5^ nm and a dwell time of 5 s per wavelength step. The acceleration voltage and beam current were set to 15 keV and 15 nA, respectively.

### Time-of-flight secondary ion mass spectrometry (ToF-SIMS)

High spatial resolution maps of ^19^F^-^, ^35^Cl^-^, ^28^Si^+^ and ^27^Al^+^ were performed using the Ga^+^ Tescan Lyra3 Focused Ion Beam Scanning Electron Microscope (FIB-SEM) equipped with a Tofwerk ToF-SIMS detector at Curtin University in Perth, Australia. An accelerating voltage of 20 kV and a current of 500 pA was used for the Ga^+^ primary ion beam. Maps of 40 x 40 μm^2^ were acquired with a pixel resolution of 40 nm. A total of 100frames were collected for the negative ion maps (^19^F^-^ and ^35^Cl^-^) and 20 frames for the positive ion maps (^28^Si^+^ and ^27^Al^+^). ToF-SIMS Explorer version 1.3 software was used for acquisition and processing of the data. Backscattered and secondary electron images were acquired with the same instrument. The electron bean was operated at 10 kV.

## Supporting information

S1 Appendix Description of DHQ-4924-H and implications on its taphonomy. (DOCX)

S1 Fig **(A)** Optical microscope transmission image of the entire sample DHQ-4924-H prior to cutting and grinding. Several flow structures can be recognized in the matrix alongside the fossil inclusions. Detailed images of **(B)** the forelimb in lateral and **(C)** medial view were taken under crossed polarized light.
(DOCX)

S2 Fig μ-CT images of sample DHQ-2924-H. **(A)** Reconstruction of the bone tissue revealed two broken parts of the forelimb (white and blue arrows). **(B)** The green areas resemble a large crack (blue arrow) as well as parts of the soft tissue. The crack cuts the radius and ulna and continues through the whole amber piece (not fully shown in B). A swelling of the soft tissue can be recognized opposite to the damaged part of the humerus, which we interpret as an edema (white arrow).
(DOCX)

S3 Fig Optical transmission images of sample DHQ-4924-H. **(A)** Exposed head of the humerus surrounded by peeled off parts of skin and air bubbles. **(B)** Large crack (black arrow) that cuts through radius and ulna (also shown in the CT-images, S2 Fig). **(C)** Skin remains and numerous bubbles within the matrix. The tiny black spots are likely sheds that formerly have been part of the integument. **(D)** Proximal part of the humerus under crossed polarized light. Note the presence of lacunae (blue arrow) which are visible in the middle part of the bone.
(DOCX)

S4 Fig Time-of-flight secondary ion mass spectrometry (ToF-SIMS) measurements of sample DHQ-4924-H. **(A)** Backscattered electron and **(B)** secondary electron images of the region around the crack. **(C-F)** Elemental maps of Silicate, Aluminium, Fluorine, and Chlorine in the region of interest shown in B.
(DOCX)
