# Supplementary material for: Fluoridation of a lizard bone embedded in Dominican amber suggests open-system behavior
Source: PLoS One. 2020 Feb 26;15(2):e0228843. doi: 10.1371/journal.pone.0228843 (PMC7043737; doi:10.1371/journal.pone.0228843)
Supplement: S1 Appendix — (DOCX) [file pone.0228843.s001.docx]

Supporting Information for

Fluoridation of a lizard bone embedded in Dominican amber suggests open-system behavior

Hans Jonas Barthel, Denis Fougerouse, Thorsten Geisler, Jes Rust

Email: [jbarthel@uni-bonn.de](mailto:jbarthel@uni-bonn.de)

S1 Appendix. Description of DHQ-4924-H and implications on its taphonomy

The amber piece DHQ-2924-H contains inclusions of a fore limb of an undetermined species of *Anolis* (Squamata, Dactyloidae) and an undetermined fairy wasp (Hymenoptera, Mymaridae, S1 Fig). The piece is built up of a succession of flows (“Schlauben”) which can be seen by naked-eye observation and were also detectable during segmentation of the CT slides. In total four major flow patterns can be identified whereas only the last two patterns contain the fossils. They are embedded within separate flows and the fore limb lies on the surface of one of these structures. The presence of “Schlauben” is important for two reasons: (i) they indicate an accumulation of resin at the surface of the stem which means (ii) that the lizard got actively into the resin because a dead lizard would not adhere to a vertical plane.

A large crack runs through the piece of amber and even cuts through the lizard limb at the proximal part of radius and ulna (S2B and S3 Figs). The crack must have been formed by the time the resin was completely hardened and represents a favored pathway for diffusional processes.

Sheds of skin are present in the matrix, especially in the region of the exposed head of the humerus S3 Fig). The peeled off parts of the skin, together with the numerous air bubbles, indicate that the resin did not harden fast after the forelimb had been trapped.

CT-scans reveal that the humerus is broken in its middle part (S2 Fig). As there is no large crack to be observed that could have caused this injury *post mortem,* it must have happened shortly before the embedding of the sample or when the resin was still viscous. This assumption is supported by the presence of a roughly ellipsoid shaped area which has a higher density than most of the other soft tissue (cf. S2B Fig). Due to its different physical properties, we interpret this structure as an edema (a local assemblage of body fluids cf. S2B Fig) especially because it is directly opposite to the damaged spot.

Taking all these observations into account, we propose the following model for our observations:

While climbing the tree, the lizard got into contact with a flow of resin and could not escape its sticky trap (note the small size of the lizard). After some time, it attracted the attention of a predator that ripped off the lizard, leaving solely the fore limb in the resin. Later on, the resin hardened and became deposited within the surrounding soil which represents the starting point of its diagenesis. The presence of “Schlauben”, the splintered humerus, the edema, the peeled off parts of the skin, and lastly the numerous air bubbles strongly support this model.
