# Supplementary material for: Fluoridation of a lizard bone embedded in Dominican amber suggests open-system behavior
Source: PLoS One. 2020 Feb 26;15(2):e0228843. doi: 10.1371/journal.pone.0228843 (PMC7043737; doi:10.1371/journal.pone.0228843)
Supplement: S1 Fig — (A) Optical microscope transmission image of the entire sample DHQ-4924-H prior to cutting and grinding. Several flow structures can be recognized in the matrix alongside the fossil inclusions. Detailed images of (B) the forelimb in lateral and (C) medial view were taken under crossed polarized light. (DOCX) [file pone.0228843.s002.docx]

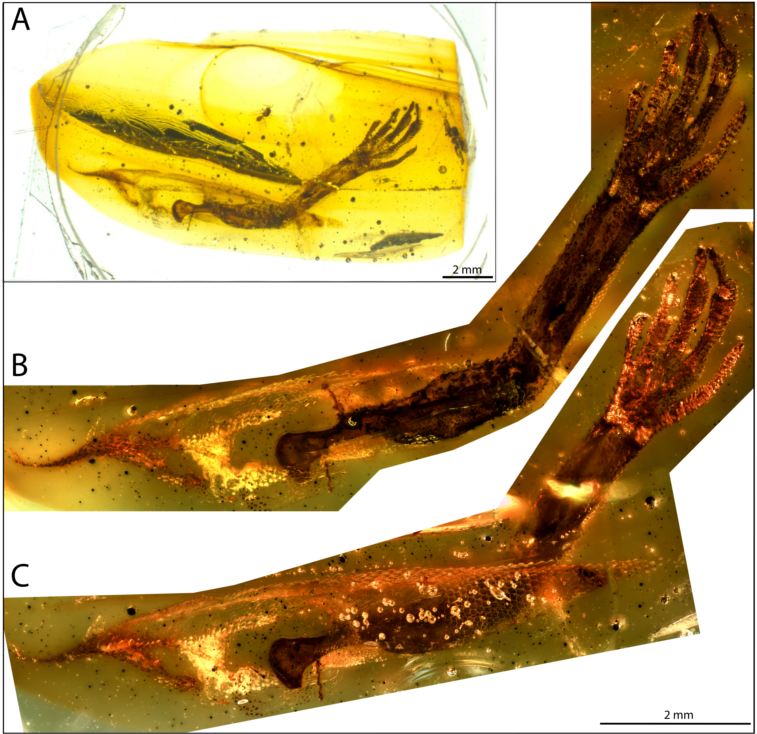


S1 Fig (A) Optical microscope transmission image of the entire sample DHQ-4924-H prior to cutting and grinding. Several flow structures can be recognized in the matrix alongside the fossil inclusions. Detailed images of (B) the forelimb in lateral and (C) medial view were taken under crossed polarized light.
