# Supplementary material for: Fluoridation of a lizard bone embedded in Dominican amber suggests open-system behavior
Source: PLoS One. 2020 Feb 26;15(2):e0228843. doi: 10.1371/journal.pone.0228843 (PMC7043737; doi:10.1371/journal.pone.0228843)
Supplement: S2 Fig — (A) Reconstruction of the bone tissue revealed two broken parts of the forelimb (white and blue arrows). (B) The green areas resemble a large crack (blue arrow) as well as parts of the soft tissue. The crack cuts the radius and ulna and continues through the whole amber piece (not fully shown in B). A swelling of the soft tissue can be recognized opposite to the damaged part of the humerus, which we interpret as an edema (white arrow). (DOCX) [file pone.0228843.s003.docx]

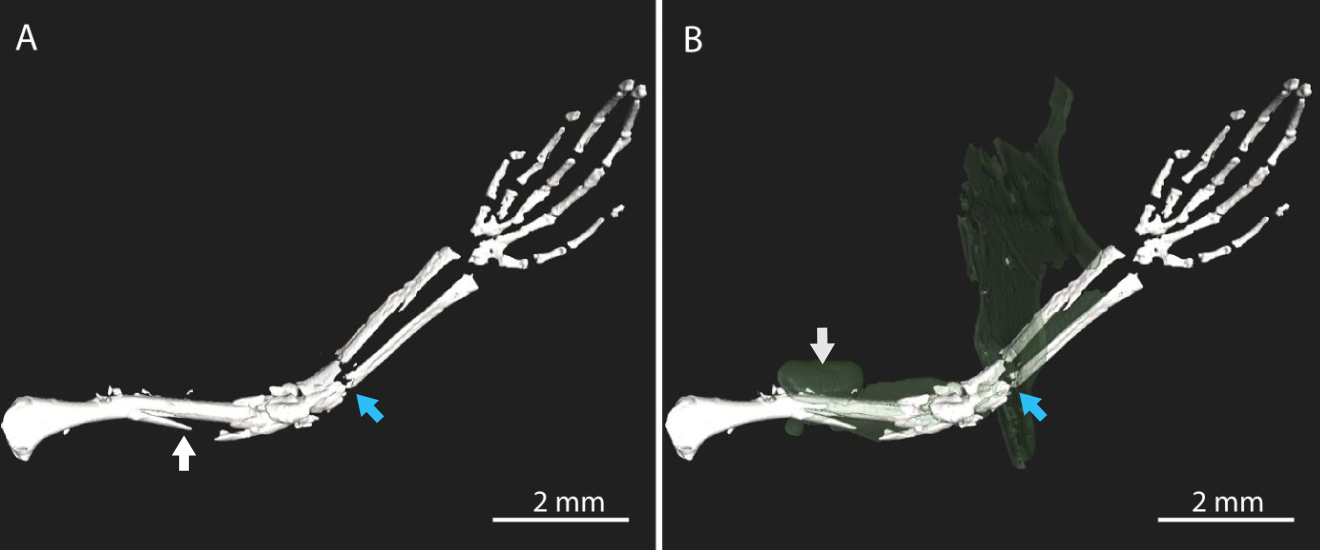


**S2 Fig** µ-CT images of sample DHQ-2924-H. **(A)** Reconstruction of the bone tissue revealed two broken parts of the forelimb (white and blue arrows). **(B)** The green areas resemble a large crack (blue arrow) as well as parts of the soft tissue. The crack cuts the radius and ulna and continues through the whole amber piece (not fully shown in B). A swelling of the soft tissue can be recognized opposite to the damaged part of the humerus, which we interpret as an edema (white arrow).
