# Supplementary material for: Fluoridation of a lizard bone embedded in Dominican amber suggests open-system behavior
Source: PLoS One. 2020 Feb 26;15(2):e0228843. doi: 10.1371/journal.pone.0228843 (PMC7043737; doi:10.1371/journal.pone.0228843)
Supplement: S3 Fig — (A) Exposed head of the humerus surrounded by peeled off parts of skin and air bubbles. (B) Large crack (black arrow) that cuts through radius and ulna (also shown in the CT-images, S2 Fig). (C) Skin remains and numerous bubbles within the matrix. The tiny black spots are likely sheds that formerly have been part of the integument. (D) Proximal part of the humerus under crossed polarized light. Note the presence of lacunae (blue arrow) which are visible in the middle part of the bone. (DOCX) [file pone.0228843.s004.docx]

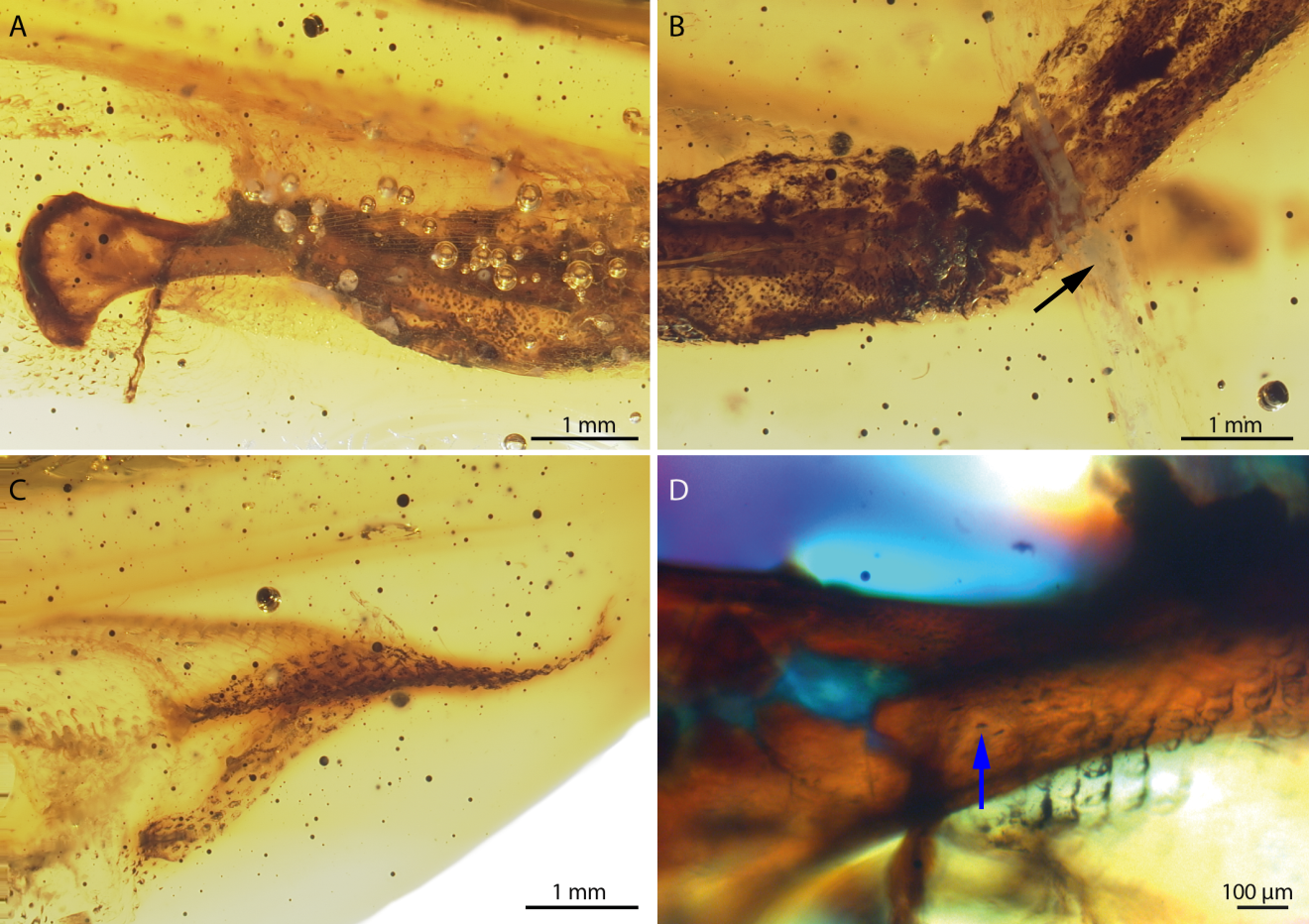


**S3 Fig** Optical transmission images of sample DHQ-4924-H. **(A)** Exposed head of the humerus surrounded by peeled off parts of skin and air bubbles. **(B)** Large crack (black arrow) that cuts through radius and ulna (also shown in the CT-images, Fig. S2.). **(C)** Skin remains and numerous bubbles within the matrix. The tiny black spots are likely sheds that formerly have been part of the integument. **(D)** Proximal part of the humerus under crossed polarized light. Note the presence of lacunae (blue arrow) which are visible in the middle part of the bone.
