# Supplementary material for: Fluoridation of a lizard bone embedded in Dominican amber suggests open-system behavior
Source: PLoS One. 2020 Feb 26;15(2):e0228843. doi: 10.1371/journal.pone.0228843 (PMC7043737; doi:10.1371/journal.pone.0228843)
Supplement: S4 Fig — (A) Backscattered electron and (B) secondary electron images of the region around the crack. (C-F) Elemental maps of Silicate, Aluminium, Fluorine, and Chlorine in the region of interest shown in B. (DOCX) [file pone.0228843.s005.docx]

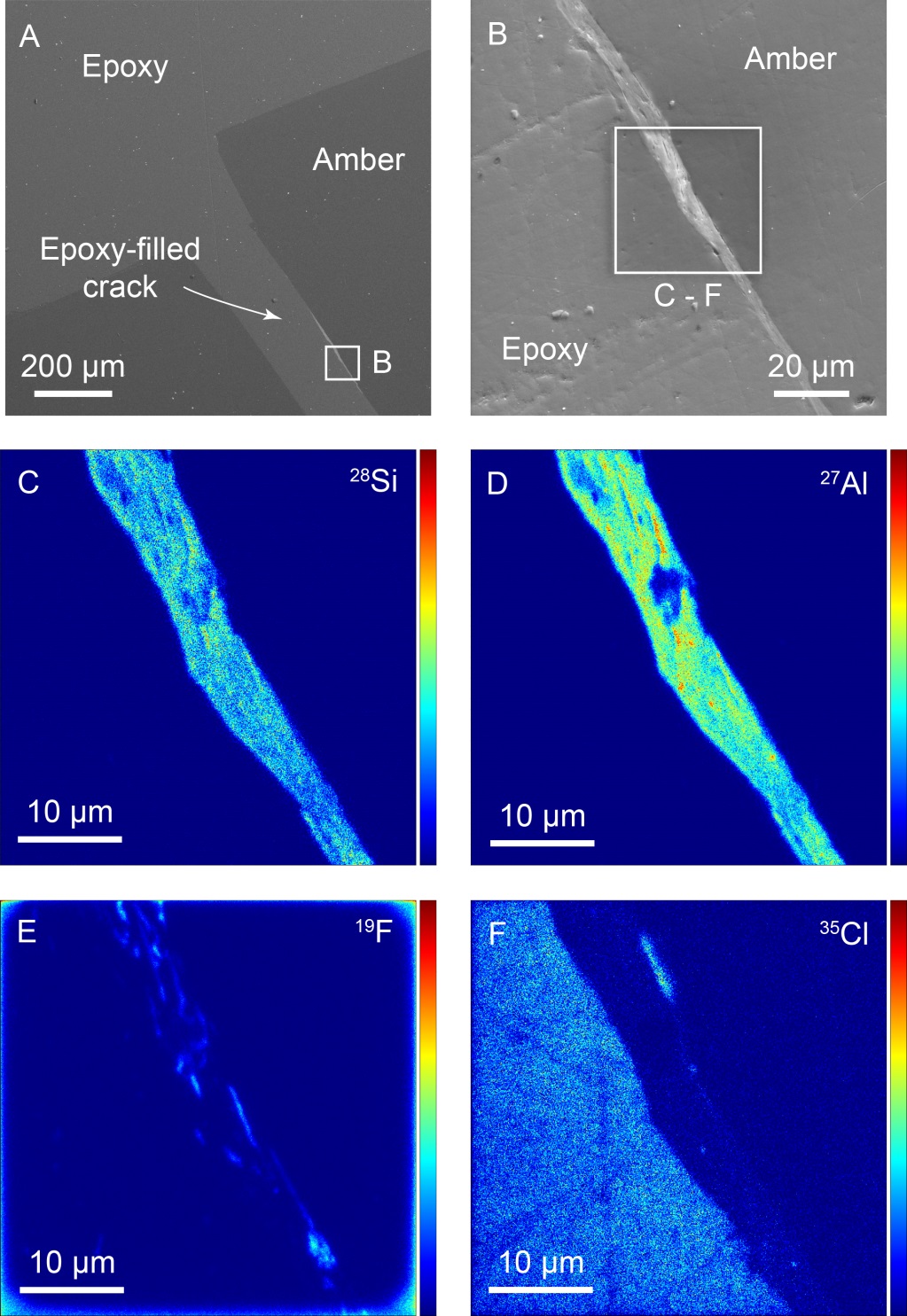


**S4 Fig** Time-of-flight secondary ion mass spectrometry (ToF-SIMS) measurements of sample DHQ-4924-H. **(A)** Backscattered electron and **(B)** secondary electron images of the region around the crack. **(C-F)** Elemental maps of Silicate, Aluminium, Fluorine, and Chlorine in the region of interest shown in B.
